# Retinoic Acid affects Lung Adenocarcinoma growth by inducing differentiation via GATA6 activation and EGFR and Wnt inhibition

**DOI:** 10.1038/s41598-017-05047-z

**Published:** 2017-07-06

**Authors:** Giovanni Zito, Flores Naselli, Laura Saieva, Stefania Raimondo, Giovanna Calabrese, Claudio Guzzardo, Stefano Forte, Christian Rolfo, Rosalba Parenti, Riccardo Alessandro

**Affiliations:** 1 Department of Biopathology and Medical Biotechnology, Biology and Genetics Section, University of Palermo, Palermo, Italy; 2 Department of Biomedical and Biotechnological Sciences, Physiology Section, University of Catania, Catania, Italy; 3 IOM Ricerca, Viagrande, Catania, Italy; 4 Phase I - Early Clinical Trials Unit, Oncology Department, Antwerp University Hospital, Antwerp, Belgium

## Abstract

A fundamental task in cancer research aims at the identification of new pharmacological therapies that can affect tumor growth. Differentiation therapy might exploit this function not only for hematological diseases, such as acute promyelocytic leukemia (APML) but also for epithelial tumors, including lung cancer. Here we show that Retinoic Acid (RA) arrests *in vitro* and *in vivo* the growth of Tyrosine Kinase Inhibitors (TKI) resistant Non Small Cell Lung Cancer (NSCLC). In particular, we found that RA induces G0/G1 cell cycle arrest in TKI resistant NSCLC cells and activates terminal differentiation programs by modulating the expression of GATA6, a key transcription factor involved in the physiological differentiation of the distal lung. In addition, our results demonstrate that RA inhibits EGFR and Wnt signaling activation, two pathways involved in NSCLC progression. Furthermore, we uncovered a novel mechanism in NSCLC that shows how RA exerts its function; we found that RA-mediated GATA6 activation is necessary for EGFR and Wnt inhibition, thus leading to 1) increased differentiation and 2) loss of proliferation. All together, these findings prove that differentiation therapy might be feasible in TKI resistant NSCLCs, and shed light on new targets to define new pharmacological therapies.

## Introduction

One of the major goals of cancer research is to identify the molecular mechanisms that can trigger tumor arrest and potential tumor regression over time. Research progresses in the past decades identified genetic and epigenetic modifications as main hallmarks of neoplastic transformations, leading to a block of normal cell differentiation coupled with uncontrolled proliferation. Currently, most of the tumors are treated with cytotoxic agents in order to induce cancer cell death. Unfortunately, over the last 50 years of treating cancer patients, we learned that conventional approaches (e.g., conventional cytotoxic agents, targeted antibodies or small molecule inhibitors) are still not sufficient in defining cures for the majority of cancer patients^1^. In addition, prolonged chemotherapy treatment in many cases leads to acquired resistance to the drugs, thus reducing the chances of patients to survive to the disease. For this reason, several attempts in the past have been tried to overcome this problem, including the tumor differentiation therapy. Differentiation therapy re-activates endogenous differentiation programs in cancer cells with subsequent loss of the tumor phenotype, mainly due to cell maturation^2^. In the past decades, a variety of agents including retinoids, histone deacetylase inhibitors (HDACI), PPARγ agonists, and others, currently in use for a variety of malignancies, have been shown to induce differentiation in solid tumors^3–6^. However, the lack of deep knowledge on the molecular mechanisms of normal cell/organ differentiation made this type of treatment quite unsuccessful, at least for most of the solid tumors. To date, tumor differentiation therapy based on Retinoic Acid (RA) treatment is the only one successfully used to treat patients with acute promyelocytic leukemia (APML)^3^. In particular, it has been showed that the combined use of RA and chemotherapy leads to 75% of complete remission in newly diagnosed APML patients^7^. Recently, our group and others began to define the molecular mechanisms mediated by RA to induce epithelial cancer differentiation in skin, breast and endometrial cancer models^8–10^.

Lung cancer is the leading major cause of death for both men and women worldwide^11^. Clinically, two main types of lung cancer are known: small cell lung cancer (SCLC, 10–15%) and non-small cell lung cancer (NSCLC, 85%), both originating from epithelial tissue of the lung structures^12^. The prognosis is extremely poor, as the majority of patients with NSCLC are in advanced stage of disease at the time of the diagnosis, and half of the patients treated initially for potentially curable early stage disease will recur with metastatic disease^13^. Recently, the identification of mutations in lung cancer led to the development of targeted therapy to improve the survival of subsets of patients with metastatic disease^14^. In particular, subsets of NSCLC, defined by specific mutations in the epidermal growth factor receptor (EGFR) gene^15^, can be treated with Tyrosine Kinase Inhibitors (TKIs), including Gefitinib, Erlotinib and Afatinib, by achieving tumor response rate of 70–80% and progression free survival of 10 to 14 months^16^. Unfortunately, patients treated with TKIs often develop a mechanism of resistance to the drug, due in most of the cases to a secondary mutation in the *EGFR* gene (T790M)^17^. In light of the above data, there is still the need to develop new therapies able to overcome the mechanisms of acquired resistance in the treatment of advanced stage NSCLC. Here we report for the first time a novel axis of signaling activation regulated by RA in NSCLC cells. In particular, we show that RA induces *in vitro* terminal differentiation in TKI resistant NSCLC cell lines by activating the transcription factor GATA6. In addition, our findings demonstrate that GATA6 directly down-regulates *EGFR* transcription and Wnt signaling activation. Finally, we show that RA treatment *in vivo* delays tumor proliferation rate in a xenograft model of NSCLC. Taken together, our results provide a molecular basis to understand mechanisms of NSCLC differentiation, thus suggesting new therapeutical approaches for the treatment of the disease.

## Results

### Retinoic Acid affects *in vitro* NSCLC cell growth

**Figure 1:**
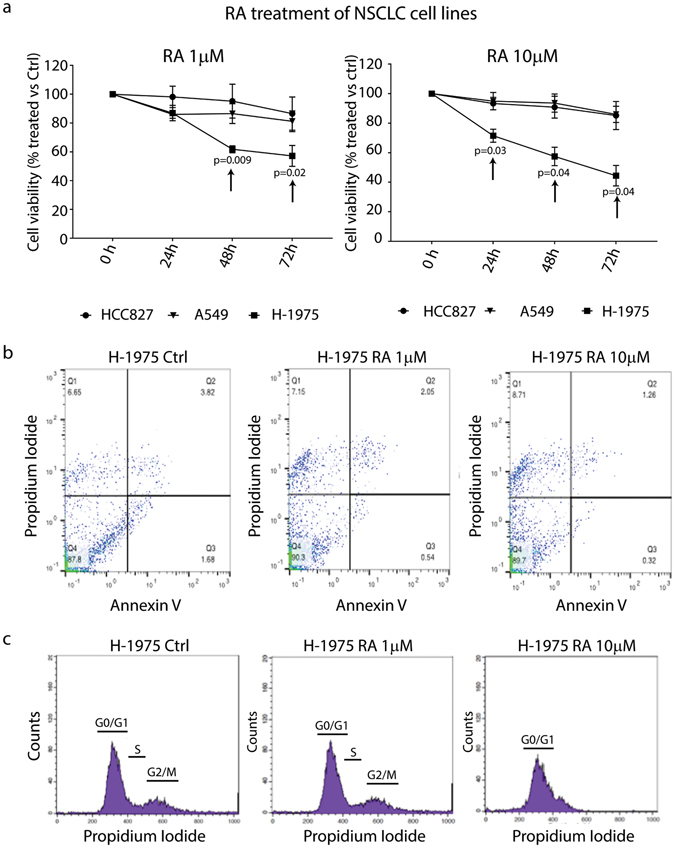
Retinoic Acid affects the growth of TKI resistant NSCLC cells. **(a)** Cell viability assay (MTT) of A549, HCC827 and H-1975 NSCLC cell lines treated with 1 and 10 µM RA for 24–48 and 72 hrs. Black arrows indicate the effect of RA on the TKI resistant H-1975 NSCLC cell line (right and left panel). Data are represented as mean ± SD and indicate the percentage of RA treated cells versus an untreated control (n = 4). **(b)** Representative Annexin V/PI assay analyzed by FACS to assess whether RA treatment induces apoptosis in H-1975 cells (n = 3). **(c)** Cell cycle analysis of H-1975 NSCLC cells treated with 1 and 10 µM RA for 72 hours (n = 4). Statistical analysis has been obtained by unpaired multiple t test.

In order to test whether RA could be used as differentiation inducer in epithelial tumors, we decided to use NSCLC cell lines as models for our studies. In particular, we used adenocarcinoma cell lines resistant or not to the action of TKIs (Gefitinib-sensitive A549 and HCC827 cells, Gefitinib-resistant H-1975 cells). First, to test how RA affects NSCLC growth, we performed cell viability assays, and interestingly, we found that RA affects the growth of TKI resistant H-1975 NSCLC cells. In particular, our analysis showed that increasing doses of RA (1 µM and 10 µM, the former representing the peak plasma levels of a single oral dose of RA^18^) arrest cell growth at 48 and 72 hours, while the same treatment did not have the same effect on A549 and HCC827 cells (Fig. 1a). To define the specificity of RA treatment in the induction of the described phenotype, we performed a co-treatment with BMS493, a pan inverse RA agonist^19^. qRT-PCR data clearly showed a down-regulation of RA target genes (*RARB*) or RA-associated genes (*CRABP2* and CYP26B1) when the cells were co-treated with different doses of RA and BMS 493 (Fig. S1a).

If RA affects the growth of H-1975 cells, we asked which was the cellular mechanism activated by the RA treatment. To test whether apoptosis occurred, we performed AnnexinV/Propidium Iodide FACS experiments. Interestingly, our results on H-1975 cells suggested that RA did not activate early apoptotic events, as showed in Figs 1b and S1b (quadrants Q2 and Q3–Ctrl vs RA treatment for 24 and 48 hrs). Notably, we found moderate apoptosis when the cells were treated with RA for 72 hrs (Fig. S1b right and left panel). Furthermore, we performed cell cycle analysis on H-1975 cells, and we found that RA (particularly 10 µM) arrested the cell cycle in the G0/G1 phase (Fig. 1c), consistent with previous reports^9^. All together, these experiments might suggest that RA affects H-1975 cell growth by arresting the cell cycle at early time points and moderate apoptosis at late ones.

### RA affects the growth of a new generated TKI resistant cell line

Given the RA specific effect to affect the growth of TKI resistant NSCLC cells, we decided to generate a new TKI resistant cell line starting from a sensitive one. For this purpose, HCC827 were subjected to increased doses of Gefitinib, as described in detail in the material and method section (Supplementary Fig. S2a). The generated cell lines HCC827R1 and HCC827R2 were treated with high doses of gefitinib (1 µM) and MTT assay was performed to check cell viability. As expected, our findings demonstrated that HCC827R1 and HCC827R2 were resistant to high doses of gefitinib, while the sensitive one was strongly affected in its viability (Supplementary Fig. S2b). Moreover, gefitinib treatment a month after the end of the selection demonstrated that, even in absence of daily TKI treatment, HCC827R1 and HCC827R2 maintained their resistant phenotype (Supplementary Fig. S2c). Finally, HCC827R1 and HCC827R2 cells were treated with RA and cell viability, as well as apoptosis, were analyzed. Our results showed that RA treatment affects the growth of both cell lines at 48 and 72 hrs, in particular when compared with HCC827 TKI sensitive cells (Supplementary Fig. S3a). However, the effect was not as strong as we found for H-1975 cells showed in Fig. 1a. Moreover, AnnexinV/PI treatment at 48 hrs did not show any sign of apoptotic events (Supplementary Fig. S3b). Taken together, these results demonstrated the specific effect of RA to affect the growth of TKI resistant cells, although it has still to be defined the biological and molecular mechanisms involved.

### RA activates differentiation programs in TKI resistant NSCLC cells

**Figure 2:**
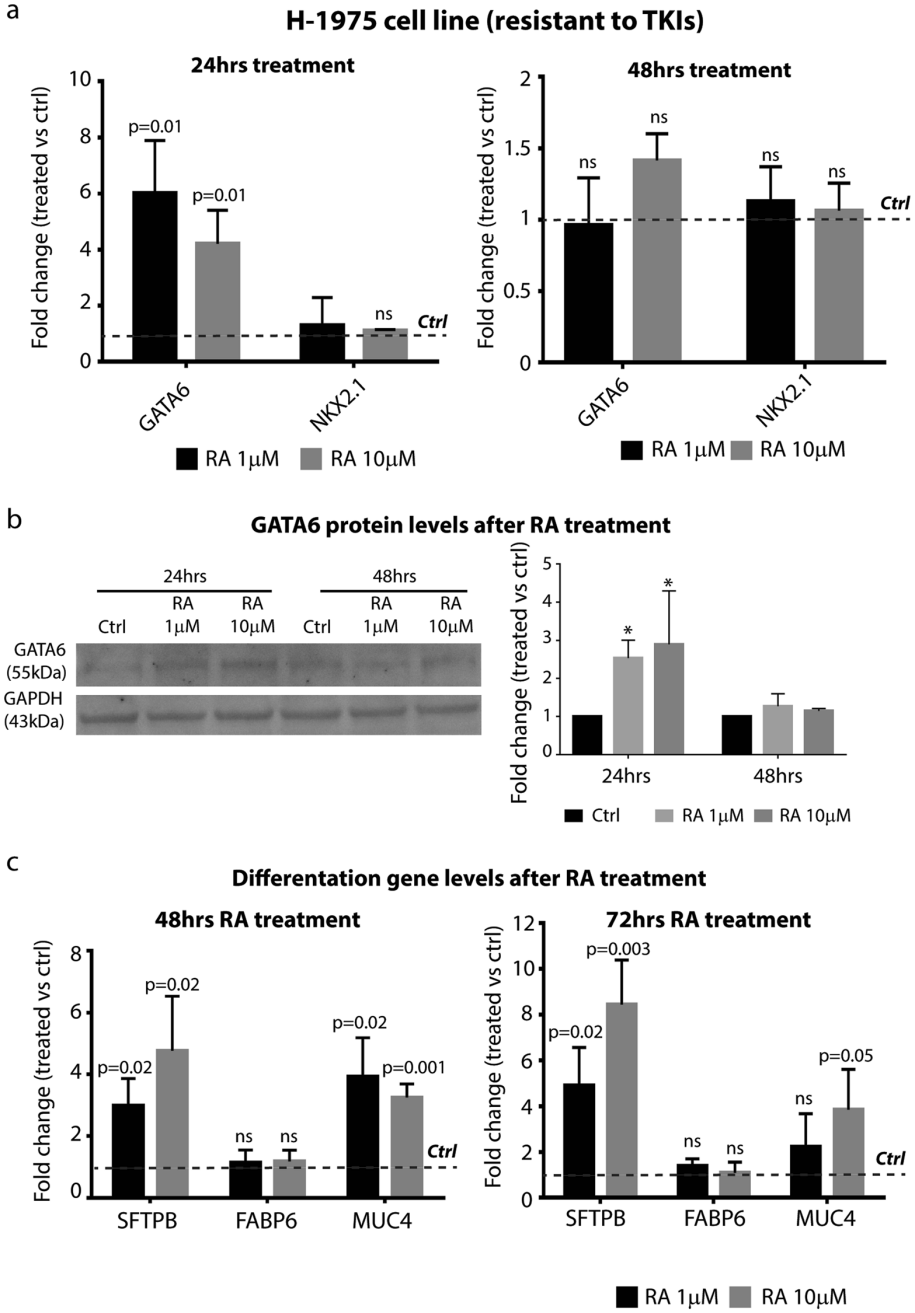
RA treatment induces TKI resistant NSCLC cell differentiation. **(a)** qRT-PCR analysis of *GATA6* and *NKX2.1* in H-1975 NSCLC cell line treated with 1 and 10 µM RA for 24 and 48 hrs. The comparison has been conducted by using the ΔΔCT method and normalized to *GAPDH* transcript. Dotted line represents the normalized expression levels of each transcript analyzed in untreated cells. Data are represented as mean ± SD. Statistical analysis has been obtained by paired t test (n = 3). **(b)** Left panel, Representative Western blotting analysis of GATA6 and GAPDH in H-1975 cells treated with 1 and 10 µM RA for 24 and 48 hrs. Right panel, densitometric analysis of GATA6 levels, normalized versus GAPDH, used as loading control (n = 4) (*=0.02). **(c)** qRT-PCR analysis of GATA6 target genes in H-1975 NSCLC cells treated with 1 and 10 µM RA for 48 and 72 hrs. The comparison has been conducted by using the ΔΔCT method and normalized to *GAPDH* transcript. Dotted line represents the normalized expression levels of each transcript analyzed in untreated cells. Data are represented as mean ± SD. Statistical analysis has been obtained by unpaired t test (n = 3).

The results described above suggest that differentiation programs might occur in TKI resistant NSCLC cells upon RA treatment. In order to verify this hypothesis, we looked at the expression of transcription factors known to initiate the differentiation cascade in normal type II alveolar epithelial cells of the distal lung, such as GATA6 and NKX2.1^20–22^. Quantitative Real-Time-PCR (qRT-PCR) analysis on RA treated H-1975 and HCC827R1 cells showed that *GATA6* is considerably up-regulated 24 hours after treatment in comparison with the untreated cells, both at 1 and 10 µM RA (Fig. 2a, left panel and Supplementary Fig. S4a). Notably, *GATA6* activation was arrested at later time points (48 hrs), thus indicating that a transient up-regulation occurs in the treated cells. Western Blotting experiments confirmed our findings, thus showing GATA6 up-regulation also at a protein level (Fig. 2b). In contrast, we found that *NKX2.1* transcription levels were not regulated by RA treatment. Finally, to define whether GATA6 activation could activate differentiation programs in NSCLC cells upon RA treatment, we screened for GATA6 target genes associated with lung terminal differentiation, including *SFPTB* and *MUC4* ^23, 24^. Consistent with our hypothesis, qRT-PCR analysis showed that *SFPTB* and *MUC4* were strongly up-regulated in TKI resistant H-1975 cells, both at 48 and 72 hours after treatment (Fig. 2c), while their up-regulation was milder in the HCC827R1 cells (Supplementary Fig. S4b). Notably, *FABP6*, that is not a differentiation marker, did not change expression upon RA treatment. All together, these results suggested that RA treatment activates terminal differentiation programs in TKI resistant cells through the positive regulation of the master transcription factor of lung differentiation GATA6.

### RA inhibits EGFR signaling *in vitro*

**Figure 3:**
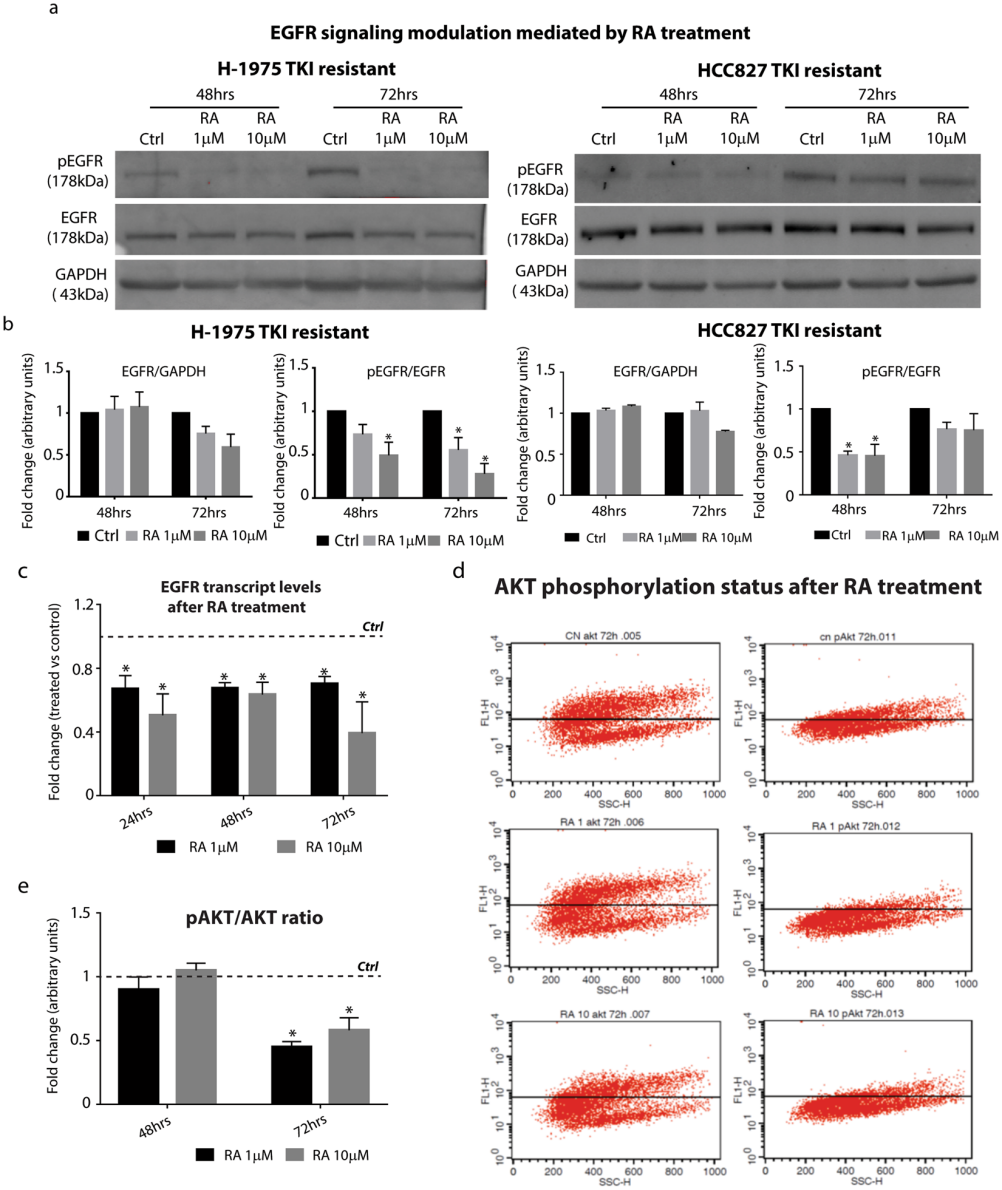
RA affects EGFR signaling activation in TKI resistant NSCLC cell line. **(a)** Representative Western Blotting analysis of EGFR and pEGFR in H-1975 and HCC827R1 cells treated with 1 and 10 µM RA for 48 and 72 hrs (n = 3). **(b)** Densitometric analysis of EGFR and pEGFR protein levels in H-1975 (left panel) and HCC827R1 (right panel) NSCLC cell lines upon RA treatment for 48 and 72 hrs. The analysis has been performed by using GAPDH as normalizer loading control (n = 3) (*=0.02). **(c)** qRT-PCR analysis of *EGFR* H-1975 NSCLC cells treated with 1 and 10 µM RA for 24, 48 and 72 hrs. The comparison has been conducted by using the ΔΔCT method and normalized to *GAPDH* transcript. Dotted line represents the normalized expression levels of each transcript analyzed in untreated cells. Data are represented as mean ± SD. Statistical analysis has been obtained by unpaired t test (n = 3) (*=0.02). **(d)** Representative FACS plot representing AKT and pAKT levels in H-1975 cells treated with RA for 72 hrs (n = 3). **(e)** Quantification analysis of pAKT levels in H-1975 cells treated with RA for 48 and 72 hrs (n = 3) (*=0.02).

Terminal differentiation does not account by itself for the TKI resistant cell phenotype described above upon RA treatment. In particular, we reasoned that increased differentiation alone could not justify the phenotype observed in the experiments showed in Fig. 1a and Supplementary Fig S3a. Thus, we hypothesized that RA negatively regulated other signaling pathways. Among them, we focused our attention on EGFR signaling, as different mutations leading to its constitutive activation have been described in a high percentage of lung adenocarcinomas^25^. In order to test whether RA treatment affected EGFR signaling, we performed Western Blotting analysis on H-1975 and HCC827R1 lysates collected after RA treatment. Interestingly, we found that RA was able to reduce EGFR protein levels, leading to a strong down-regulation of the activated form pEGFR (Fig. 3a right and left panel). In particular, the densitometric analysis of EGFR and pEGFR, normalized by using GAPDH as loading control, revealed that while EGFR protein levels started to be down-regulated at 72 hours, pEGFR levels were already modulated at 48 hrs (Fig. 3b, right and left panel). These findings suggested that EGFR and pEGFR down-regulation could depend on different mechanisms occurring upon RA treatment. In order to test whether EGFR inhibition is regulated at a transcriptional levels, mRNA from H-1975 cells RA-treated was subjected to qRT-PCR for the detection of EGFR transcript. Interestingly, our results clearly show that RA treatment affects the *EGFR* transcription at all the time points analyzed (24 to 72 hrs, Fig. 3c). Finally, to prove that EGFR signaling pathway was switched off in H-1975 cells, we checked AKT phosphorylation status in the cells upon RA treatment. FACS experiments on total and phosphorylated AKT levels demonstrated that RA treatment reduces pAKT, specifically with a 50% reduction at 72 hrs with the two RA doses (1 and 10 µM, Fig. 3d,e). These findings might suggest that RA directly regulates EGFR transcription and the activation of the signaling pathway.

### RA down-regulates Wnt signaling activation in TKI resistant H-1975 cells

**Figure 4:**
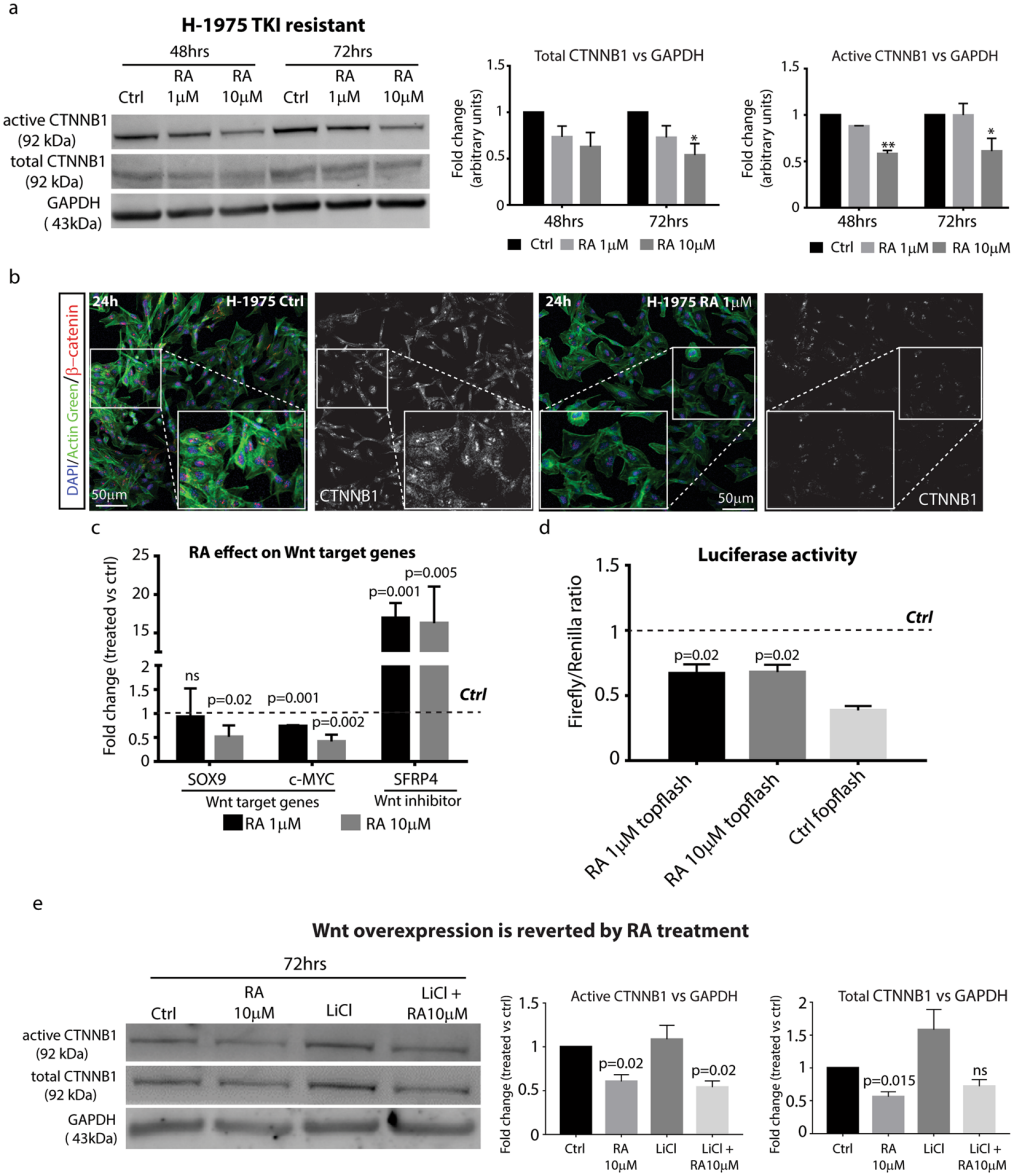
Wnt signaling activation is inhibited by RA in TKI resistant NSCLC cell line. **(a)** Left panel, representative Western Blotting analysis of total and active-CTNNB1 in H-1975 treated with 1 and 10 µM RA for 48 and 72 hrs (n = 3). Right panel, densitometric analysis of total and active-CTNNB1 protein levels in H-1975 cells upon RA treatment for 48 and 72 hrs. The analysis has been performed by using GAPDH as normalizer loading control (n = 3) (*=0.02, **=0.05). **(b)** Immunofluorescence staining of CTNNB1 in H-1975 cells treated with or not with 1 µM RA for 24 hrs (see also insets for all the experimental conditions). Cells are counterstained with Actingreen 488 (green label) and nuclei are marked in blue with DAPI (scale bar 50 µm, n = 3). **(c)** qRT-PCR analysis of Wnt target genes and the Wnt inhibitor *SFRP4* in H-1975 cells treated with 1 and 10 µM RA for 48 hrs. The comparison has been conducted by using the ΔΔCT method and normalized to *GAPDH* transcript. Dotted line represents the normalized expression levels of each transcript analyzed in untreated cells. Data are represented as mean ± SD. Statistical analysis has been obtained by unpaired t test (n = 3). **(d)** Luciferase assay of H-1975 cells transfected with Wnt reporters TOPFlash/FOPFlash and treated with 1 and 10 µM RA for 48 hrs. Data are represented as mean ± SD. The histograms represent the value obtained by calculating the Firefly/Renilla ratio, the latter used to measure transfection efficiency. Dotted line represents the normalized expression levels of luciferase activity in H-1975 untreated cells. Statistical analysis has been obtained by unpaired t-test (n = 3). **(e)** Representative Western Blotting analysis of total and active-CTNNB1 protein levels in H-1975 cells treated with 10 µM RA, 2.5 mM LiCl or co-treated (10 µM RA + 2.5 mM LiCl) for 72 hrs and its relative densitometric analysis (central and right panel). The analysis has been performed by using GAPDH as normalizer loading control (n = 3).

Recent studies showed that RA is able to affect Wnt signaling in different tumor models. In particular, we previously demonstrated that RA signaling activation leads to the spontaneous regression of Keratoacanthoma skin tumors by inhibiting Wnt pathway and stimulating terminal differentiation^8^. In addition, several evidences in the past clearly showed that Wnt signaling activation is required for NSCLC growth and metastatic progression^26, 27^. For all the findings described above, we reasoned that RA could affect Wnt activation, along with EGFR inhibition, thus justifying NSCLC tumor growth arrest. In order to evaluate this hypothesis, we measured the levels of total and active CTNNB1 (a hallmark of activated Wnt pathway) in H-1975 cells upon RA treatment. Our results showed in Fig. 4a clearly demonstrated that RA treatment down-regulated not only CTNNB1 total levels, but also the non-phosphorylated active form. The densitometric analysis suggested a time and dose-dependent CTNNB1 down-regulation at 48 and 72 hours at 1 and 10 µM RA. If CTNNB1 was down-regulated, then we reasoned that also its nuclear levels could be negatively regulated by the treatment. To address this question, we decided to perform immunofluorescence stainings on H-1975 cells upon RA treatment and checked by confocal microscopy CTNNB1 localization within the cells. Our results showed a reduction in CTNNB1 expression in H-1975 cells treated with RA, when compared with the untreated control (Fig. 4b). In particular, we observed a strong reduction in the number of nuclear CTNNB1^+^ cells. These results suggested that the loss of CTNNB1^+^ cells might account for Wnt inhibition in H-1975 cells upon RA treatment. To test this hypothesis, we screened by qRT-PCR the expression of the Wnt target genes *SOX9* and *c-MYC*. We found that RA down-regulated the expression levels of such target genes, in a dose and time-dependent manner (Fig. 4c). Interestingly, we demonstrated that RA treatment strongly up-regulated the Wnt inhibitor *SFRP4*, consistently with previous reports on skin and liver cancer^8, 28^. To functionally address whether RA treatment inhibits Wnt activation, we performed Luciferase assay analysis by using the Wnt reporter TOP-Flash system^29^. Consistent with our previous findings, we found that H-1975 cells treated with RA reduced the luciferase activity, thus confirming an effect of RA on the activation of Wnt pathway (Fig. 4d). Finally, we found that RA revert Wnt signaling activation mediated by Lithium Chloride (LiCl) treatment (Fig. 4e, right and left panel). All together, these data sustain the hypothesis that RA affect NSCLC growth not only by inducing terminal differentiation, but also by inhibiting the two proliferation pathways EGFR and Wnt.

### RA affects *in vivo* NSCLC xenograft tumor growth by inhibiting EGFR and Wnt signaling

**Figure 5:**
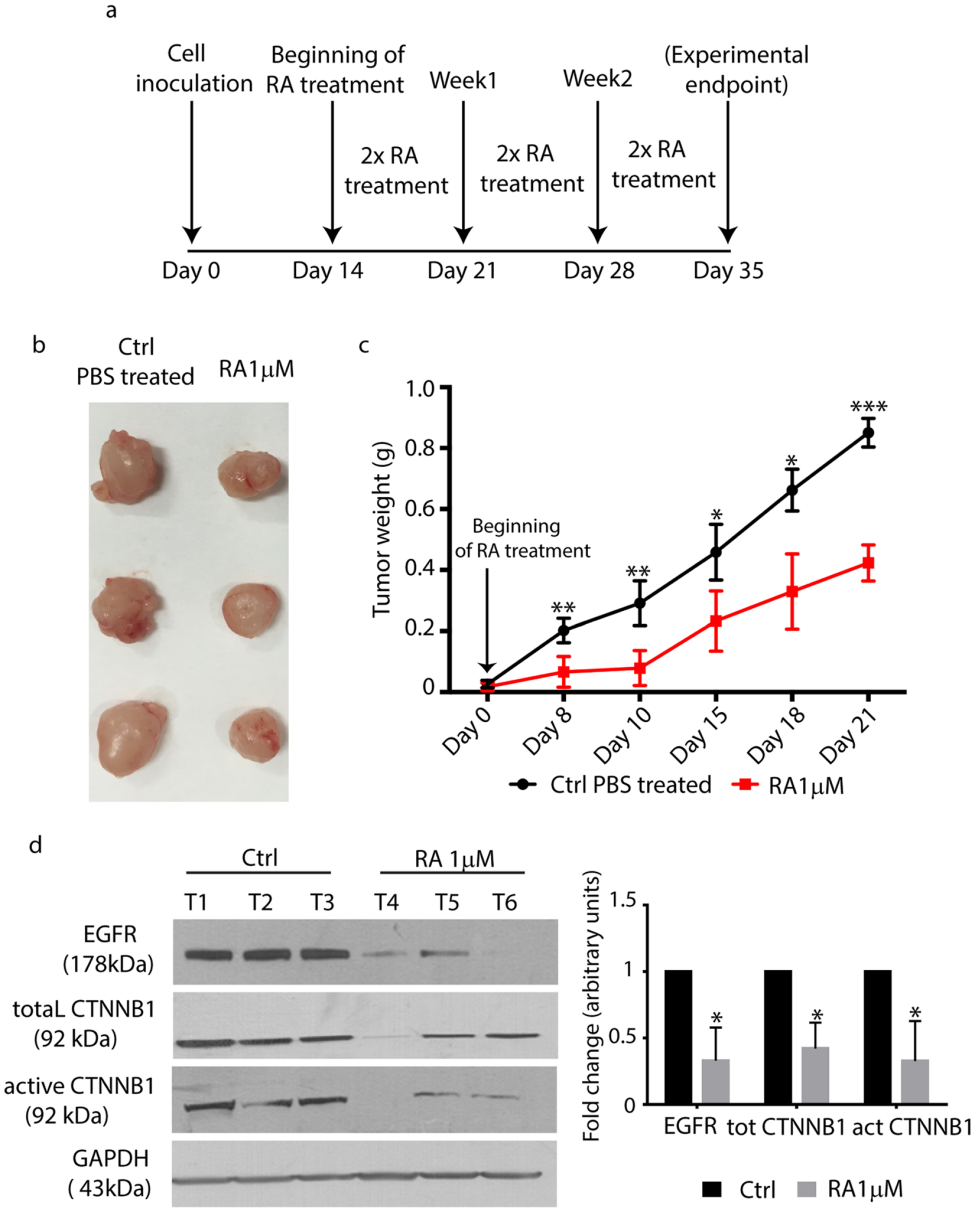
*In vivo* effects of RA on H-1975 xenografts. **(a)** Schematic representation of the *in vivo* experiment performed by injecting H-1975 cells in athymic nude mice. **(b)** Representative micrographs obtained after tumor harvesting (PBS treated vs 1 µM RA treated mice). **(c)** Tumor weight analysis of PBS and 1 µM RA treated mice. Tumor size was measured at the time points indicated in the *x* axis. Data are represented as mean ± SD (*=0.02, **=0.05, ***=0.002, obtained by unpaired t test analysis, n = 5). **(d)** Left panel, Western Blotting of EGFR, GAPDH total and active- CTNNB1 from biopsies taken from PBS and 1 µM RA treated mice. Right panel, densitometric analysis of EGFR, total and active CTNNB1 protein levels in tumor biopsies from PBS and 1 µM RA treated groups. The analysis has been performed by using GAPDH as normalizer loading control (n = 3 for each group).

To validate our *in vitro* findings, we tested *in vivo* the effect of RA on NSCLC xenograft tumors in immunodeficient athymic nude mice. H-1975 cells were injected subcutaneously into the left flank of the animals, and 2 weeks after the injection, when the tumors started to be palpable, 1 µM RA or PBS were administered intratumorally twice/week until the experimental endpoint (Fig. 5a). Our results showed that RA treatment delayed tumor growth in comparison with PBS alone treated tumors used as control (Fig. 5b and c). In particular, we found that tumor growth was arrested in the first 10 days of RA treatment, while in the next time points we observed a slight growth of the xenografts. However, Fig. 5c showed that the growth rate is still slower and significantly different from the control tumors. All together, these data suggested that RA affects tumor growth *in vivo* by delaying the proliferation rate at the first stages of tumor progression. In order to define the molecular mechanisms affected by RA treatment *in vivo*, we decided to screen for the expression and activation of EGFR and Wnt signaling pathways. Western Blotting analysis performed on tumor lysates at the end of the experimental regimen showed that EGFR protein was lost in RA-treated xenografts (Fig. 5d). In a similar manner, we found that total and active CTNNB1 were dramatically reduced in their expression, as confirmed by the densitometric analysis performed using GAPDH as loading control (Fig. 5d). Taken together, these findings clearly confirmed our *in vitro* data, thus suggesting that RA treatment arrests xenograft growth by down-regulating EGFR protein and by inhibiting Wnt/ CTNNB1 activation.

### GATA6 regulates H-1975 tumor cell arrest via EGFR and Wnt down-regulation

**Figure 6:**
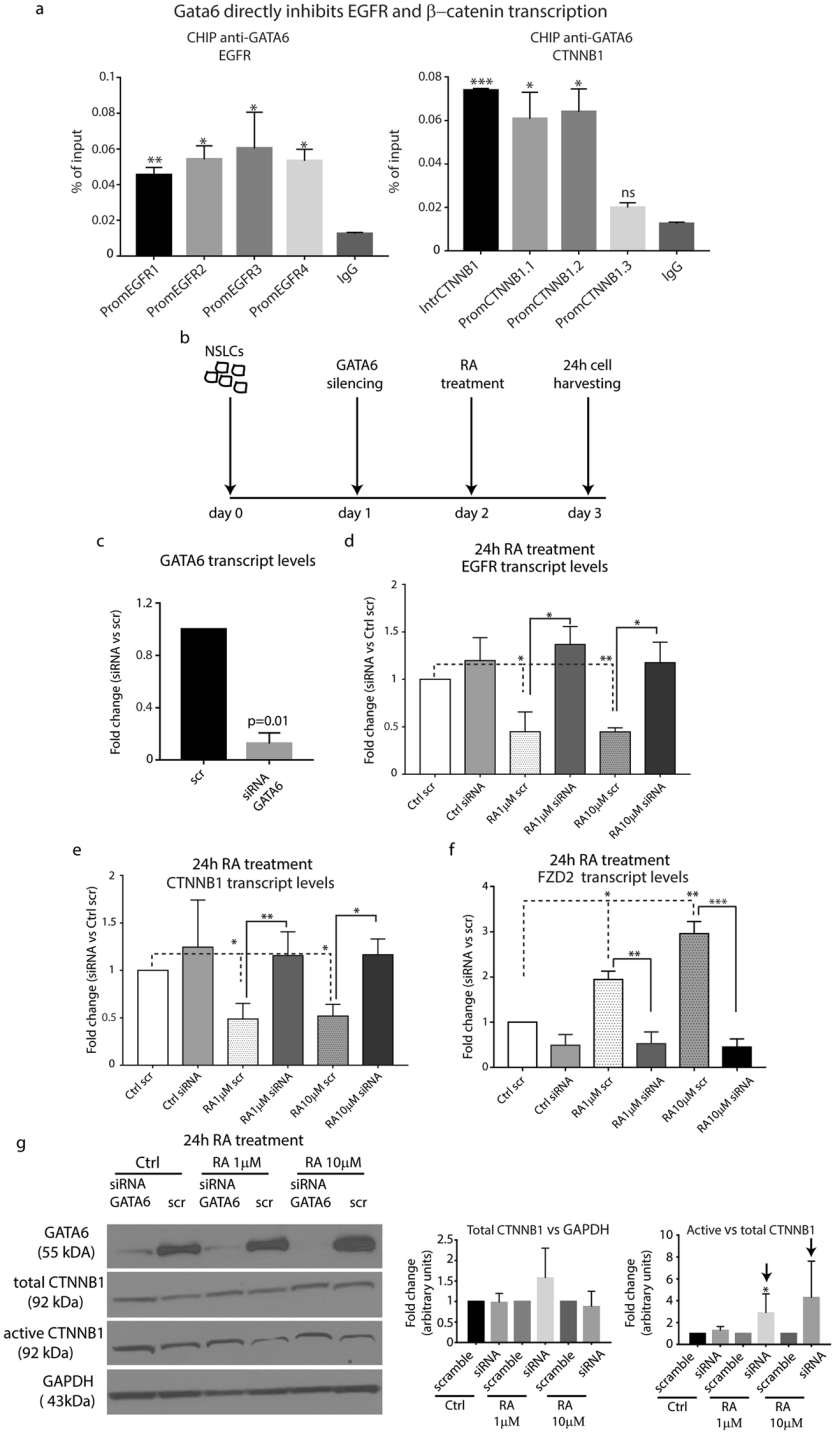
GATA6 knockdown restores *EGFR* transcription and Wnt activation. **(a)** CHIP analysis of EGFR and CTNNB1 promoters after chromatin immunoprecipitation with anti-GATA6 antibody. Signals obtained from each immunoprecipitation were expressed as a percent of the total input chromatin (see material and method section for further details) (n = 2, *=0.02, **=0.05, ***=0.002). **(b)** Schematic representation of *GATA6* silencing and RA treatment experiment in H-1975 cells. **(c)** qRT-PCR analysis of *GATA6* transcript after *GATA6* silencing. The comparison has been conducted by using the ΔΔCT method and normalized to *GAPDH* transcript. Data are represented as mean ± SD. Statistical analysis has been obtained by unpaired t-test (n = 3). **(d**,**e)** qRT-PCR analysis of *EGFR* and *CTNNB1* transcript level after *GATA6* silencing and 1 or 10 µM RA treatment for 24 hrs. The comparison has been conducted by using the ΔΔCT method and normalized to *GAPDH* transcript. The fold changes were obtained as ratio of each mRNA sample versus the Ctrl scrambled. Data are represented as mean ± SD (*<0.05, **<0.001, obtained by unpaired t test analysis, n = 3). **(f)** qRT-PCR analysis of *FZD2* transcript after *GATA6* silencing and 1 or 10 µM RA treatment for 24 hrs. The comparison has been conducted by using the ΔΔCT method and normalized to GAPDH transcript. Dotted line represents the normalized expression levels of each transcript analyzed in scramble transfected treated/untreated cells. Data are represented as mean ± SD. Statistical analysis has been obtained by unpaired t-test (n = 3). **(g)** Left panel, representative Western blotting analysis of GATA6, total and active CTNNB1 in H-1975 cells after transfection with siRNA GATA6 or scramble and treated or not with 1 and 10 µM RA for 24 hrs. Right panel, densitometric analysis of total and active-CTNNB1 levels, normalized versus GAPDH or total CTNNB1, the former used as loading control represented as mean ± SD (n = 3).

Our results suggested a role for GATA6 to induce terminal differentiation and growth arrest in TKI resistant NSCLC cells, by down-regulating EGFR and Wnt signaling activation. Specifically, the results showed in Figs 3a and 4a suggested a direct role of GATA6 to inhibit *EGFR* and *CTNNB1* transcription. In order to test this hypothesis, we performed a Chromatin Immunoprecipitation Assay (CHIP). For this purpose, we designed primers that recognized GATA6 consensus sequences (A/T/C)GAT(A/T)(A) in the *EGFR* and *CTNNB1* promoter (Supplementary Table S1). qRT-PCR analysis on the GATA6 immunoprecipitated chromatin clearly showed that GATA6 bound to *EGFR* and *CTNNB1* promoter. Specifically, we found that GATA6 binds to all the consensus sequences of the *EGFR* promoter, while it binds to three out of the four consensus sequences analyzed in the CTNNB1 promoter/first intron (Fig. 6a).

Recently, it has been showed that GATA6 inhibits Wnt signaling activation by inducing the transcription of the Wnt antagonist *FZD2* in the distal epithelium of the lung. Thus, our findings, along with the described discoveries by other groups, led us to hypothesize that *GATA6* up-regulation mediated by RA could modulate TKI resistant cell phenotype by directly inhibiting *EGFR* transcription and Wnt signaling activation. To test this hypothesis, we knock down (KD) *GATA6* in H-1975 cells, followed by RA treatment for 24 hours (Fig. 6b). *GATA6* down-regulation in siRNA-transfected cells was confirmed by qRT-PCR (Fig. 6c). To assess whether GATA6 KD altered *EGFR* transcript levels, we performed a qRT-PCR on KD and scrambled transfected H-1975 cells upon RA treatment. Consistent with our previous data, *EGFR* mRNA was down-regulated in scrambled transfected/RA treated cells (Fig. 6d). Interestingly, *EGFR* transcript levels were significantly rescued in *GATA6* KD/RA treated cells in comparison with the scrambled transfected/RA treated ones (Fig. 6d). In particular, our findings showed that 24 hours post RA treatment, *EGFR* mRNA was restored to the levels of the scrambled untreated cells. These results confirmed that GATA6 regulated directly *EGFR* transcription, and begin to shed light on the mechanisms of RA-mediated EGFR inhibition. In order to assess whether GATA6 affects Wnt signaling activation, we performed qRT-PCR analysis on the transcription levels of *CTNNB1* and *FZD2*. Our results in Fig. 6e showed that the expression levels of *CTNNB1* were restored in *GATA6* KD/RA treated cells in comparison with the scrambled transfected/RA treated ones. These results further confirmed a direct link between GATA6 and CTNNB1 transcript down-regulation. In addition, we found that RA treatment induces *FZD2* up-regulation, and that *GATA6* KD down-regulated its transcription, thus confirming previous findings^30^ (Fig. 6f). These results suggested that GATA6 might regulate Wnt activation at different levels, (1) by down-regulating *CTNNB1* transcript, and (2) by activating the transcription of Wnt inhibitors. Finally, we performed Western Blotting analysis to evaluate Wnt activation levels in KD H-1975 cells. Interestingly, we showed that *GATA6* KD/RA treated cells increased active- CTNNB1 levels compared to the scrambled transfected/RA treated control cells (Fig. 6g). In particular, the densitometric analysis in Fig. 6g, right panel, showed that active-CTNNB1 levels were restored to the levels of the scramble transfected/untreated cells (see black arrows). Furthermore, qRT-PCR analysis of Wnt target genes SOX9 and c-MYC upon GATA6 silencing and RA treatment showed a rescue of Wnt signaling activation (Supplementary Fig. S5). Taken together, these data demonstrate that GATA6, along with its major function in activating terminal differentiation programs in H-1975 cells, plays also a crucial role in (1) blocking *EGFR* and *CTNNB1* transcription, and (2) inhibiting Wnt signaling activation.

## Discussion

A major challenge in cancer biology is to define new therapeutical approaches that can increase the survival rate of the patients. Tumor differentiation therapy has been studied for long time in the past as a possible approach to overcome chemotherapic side effect, however the results obtained were difficult to interpret. First, the lack of deep knowledge on the precise molecular mechanisms driving normal cell differentiation made this type of therapy quite complicated to pursue. Second, it was difficult to envision how differentiation therapy could restore a benign phenotype in tumor cells with immutable genetic level mutation driving cancer initiation and progression. However, recent studies sustained the hypothesis that tumor differentiation might be possible in solid tumors. Dow and collaborators demonstrated that *APC* restoration in colon cancer cells could revert the malignant phenotype to functional differentiated intestinal units, thus inducing regression in tumors harboring *KRAS* ^*G12D*^ and *TRP53* mutations^31^. In addition, our group showed that (1) spontaneous regression of murine cutaneous Keratoacanthoma skin tumor is mediated by terminal differentiation induced by RA treatment, and (2) ectopic RA treatment of skin squamous cell carcinomas led to tumor regression by inhibiting Wnt signaling and activating differentiation programs^8^. All together, these findings clearly suggested that differentiation therapy might be feasible for the treatment of epithelial tumors, although it is important to identify the ideal differentiating agent to use according to the tumor genetic background.

While RA signaling was showed to induce cellular senescence in different cell types^32^ it is mainly involved in regulating the differentiation of several citotypes at early stages of organogenesis^33–36^, including the development of the alveolar tree in the fetal lung^37^. Thus, RA was used to induce tumor differentiation, and the most successful results were obtained in the treatment of APML patients^7^. In the present study, we demonstrate that RA affects the growth of NSCLC cell lines, by arresting the cells at G0/G1 phase of the cell cycle, thus inducing the activation of terminal differentiation programs. Interestingly, our data show that RA acts preferentially on TKI resistant NSCLC cell lines, with very little effects on the TKI sensitive ones. To our knowledge, these findings are quite novel for an epithelial tumor model, while the correlation between RA and TKI resistance has been proven already for Chronic Mielogenous Leukemia (CML)^38^. Recently, it was showed that TKI resistant and TKI sensitive NSCLC cell lines are molecularly different. In particular, different groups demonstrated that TKI resistant NSCLC cells showed AXL activation and up-regulation of Hippo signaling when compared with the sensitive counterpart^39, 40^. In addition, Park and collaborators suggested that IGFR1 signaling pathway is up-regulated in TKI resistant NSCLC cell lines, with the concurrent loss of *IGFBP3* ^41^. These findings clearly suggest that understanding the molecular mechanisms that differentially mediate RA effect on TKI resistant and sensitive NSCLC cell lines is crucial to define new therapies specifically directed towards TKI resistant NSCLC tumors.

In the past years, it has been showed that two major transcription factors, GATA6 and NKX2.12.1 (TTF-1) play a pivotal role in regulating the differentiation of the distal epithelium during lung morphogenesis^42, 43^. Interestingly, RA positively regulates GATA6 expression, thus confirming the activation of differentiation programs in TKI resistant NSCLC cells. While RA-mediated GATA6 up-regulation was already showed to regulate macrophages differentiation and polarization^44^, very little is known about the precise molecular mechanisms behind this phenotype.

**Figure 7:**
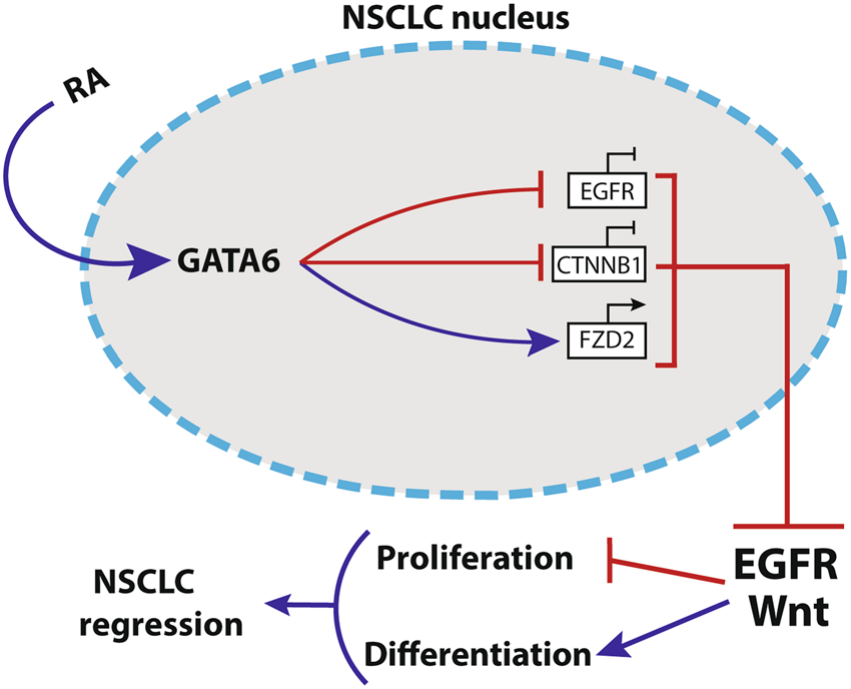
A working model of the signaling mechanisms regulated by RA to induce NSCLC regression. RA treatment increases GATA6 expression levels in differentiated NSCLC cells. GATA6 acts (1) by inhibiting *EGFR* and CTNNB1 transcript, and (2) by activating the expression of the Wnt inhibitor *FZD2*. These molecular effects lead to the down-regulation of EGFR and Wnt signaling pathways, by arresting tumor cell proliferation and promoting cell differentiation.

RA-mediated terminal differentiation does not account by itself for the NSCLC regression we observed *in vivo*. Thus, we thought that RA might regulate other molecular mechanisms driving NSCLC progression. It is well known that (1) different genetic mutations in EGFR gene are described in a high percentage of NSCLC patients worldwide^45^, and (2) EGFR constitutive activation is required for lung adenocarcinoma progression^25^. In accordance with previous reports^46, 47^, our results clearly show that RA inhibits EGFR signaling, by reducing *EGFR* transcript and down-regulating the active pEGFR protein levels. Specifically, we demonstrate that GATA6 directly binds to *EGFR* promoter. Our finding are in accordance with recent studies that demonstrated that Gata6 represses *Egfr* transcription in a pancreatic model of murine ductal adenocarcinoma^48^, thus inducing acinar differentiation. By using siRNA technology, we demonstrate that GATA6 regulates *EGFR* transcription in TKI resistant NSCLC cells. Taken together, these findings are quite novel as they provide for the first time to our knowledge the precise mechanisms by which RA exploits its function in NSCLC adenocarcinoma model (Fig. 7).

NSCLC progression and metastasization is mediated by Wnt signaling^49^, thus the inhibition of this pathway is key to define new therapies to arrest NSCLC growth. We found that RA down-regulates Wnt activation, by reducing the levels of active-CTNNB1. In addition, we clearly demonstrate that Wnt inhibition is regulated by GATA6-mediated (1) *FZD2* transcription^50^ and (2) CTNNB1 transcriptional down-regulation. Our data are consistent with several other studies showing that RA inhibits Wnt signaling in different tumor model^8, 28, 51^; however, we show for the first time to our knowledge the molecular mechanisms by which RA exerts its function in NSCLC^52, 53^.

Taken together, our data begin to shed lights on the molecular mechanisms that drive RA-mediated NSCLC differentiation, and sustain the hypothesis that differentiation therapy might be feasible for the treatment of aggressive epithelial tumors. Furthermore, our results open to the possibility to define new therapeutical strategies that could overcome TKI resistance in NSCLC.

## Material and Methods

### Ethics Statement

The study was conducted in compliance with Italian and European laws concerning animal experiments. The research protocol was authorized by Italian Ministry of Health (2014/01/13) according to Legislative Decree 116/92 and was performed according to Legislative Decree 26/2014.

### Cell culture and reagents

The human NSCLC cell lines H-1975 and HCC827 were provided by Prof. Rolfo, University of Antwerp. A549 human adenocarcinoma cell line was purchased from ATCC (ATCC® CCL-185™). Cell lines were cultured in RPMI 1640 medium (Euroclone, Milan, Italy) supplemented with 10% heat-inactivated fetal bovine serum (FBS), 100 U/ml penicillin, and 100 μg/ml streptomycin (Euroclone) and were cultured at 37 °C in a humid incubator with 5% CO_2_. Retinoic Acid (RA) and Lithium chloride (LiCl) were purchased from Sigma Aldrich (Saint Louis, MO, USA), Gefitinib and Doxorubicin were purchased from Selleckchem (Houston, TX, USA), BMS493 was purchased from Santa Cruz Biotechnology (Santa Cruz, CA, USA).

### Generation of NSCLC TKI resistant cells

To generate NSCLC TKI resistant cells, HCC827 TKI sensitive parental cells were grown in increasing doses of the tyrosine kinase inhibitor Gefitinib. Specifically, HCC827 cells were treated with Gefitinib with a starting concentration of 10 nM (Fig. S2b). Gefitinib concentration was increased usually every two weeks, once the cells began to adapt to new drug concentration. The experimental endpoint was fixed to 1 μM Gefitinib. Cells were maintained in 1 μM Gefitinib for one month, and then the drug was removed in order to verify TKI resistance.

### Viability assay

Cell viability on NSCLC cell lines was evaluated with Thiazolyl blue tetrazolium bromide (MTT, M5655-1G, Sigma Aldrich) assay^54^.

### Annexin V, cell cycle and AKT analysis

Apoptosis was detected in flow cytometry by double staining with Annexin V/propidium iodide (PI). Specifically, H-1975 cells and HCC827 TKI resistant cells were seeded into 6-well plates and after an overnight incubation, they were treated with RA (1 μM and 10 μM) or Doxorubicin 0,5 μM. After 24 and 48 h incubation, apoptosis assay was performed. Cell were incubated with 5 μl of Annexin V-FITC (BD-Biosciences, Muntan View, CA, USA) and 5 μL of PI (20 μg/ml, Sigma Aldrich) at room temperature in the dark for 15 min.

For the cell cycle assay, RA-treated cells were harvested and re-suspended in ice-cold PBS. Cold 100% Ethanol has been added drop by drop to each sample, and then the cells were incubated for 30 min on ice. At the end of the incubation, the cells were washed with PBS/1% BSA and then re-suspended in a solution containing 50 µg/ml PI. Cells were incubated for 1hr in the dark.

The level of AKT and pAKT were detected in flow cytometry by using LEUCOPERM^TM^ reagents from AbD Serotec (Raleigh, NC, USA). Specifically, H-1975 cells were seeded into 6-well plates and after an overnight incubation, they were treated with RA 1 μM and 10 μM. According to the manufacturer’s protocol, cells were fixed with Reagent A and then permeabilized with reagent B. Finally, cells were incubated 30 min at room temperature with unconjugated primary antibody (anti-AKT #9272 1:50 or anti-pAKT #4060 1:100 from Cell Signaling) and after washing with PBS/BSA cells were incubated with the FITC-conjugated secondary antibody (Life Technologies, Carlsbad, CA, USA).

For all the experiments described, stained cells were acquired on FACSCalibur^TM^ (BD-Biosciences) and analyzed by using the FlowJo software (LLC) or the BD CellQuest Pro^TM^.

### RNA extraction and qRT-PCR

Cells were seeded in 6-well plates and treated with RA at different time points. RNA was extracted using the Illustra RNAspin Mini Isolation Kit (GE Healthcare, UK), according to manufacturer’s instructions. Total RNA (1 μg) was reverse-transcribed to cDNA using the High Capacity cDNA Reverse Transcription Kit (Applied Biosystem). RT-QPCR was performed in 48-well plates using the Step-One Real-Time PCR system (Applied Biosystem).

For quantitative SYBR®Green qRT-PCR, reactions were carried out in a total volume of 20 μl containing 2 × SYBR®Green I Master Mix (Applied Biosystems), 1 μl cDNA and 1 μl of 10 μM forward and reverse primers. Primers sequences are listed in Supplementary Table S2.

### Western blotting and antibodies

SDS-PAGE Electrophoresis and Western Blotting were performed as previously described^54^. The primary antibodies used were as follows: EGFR (#2232, 1:1000), pEGFR (#3777, 1:800), active β-catenin (#8814, 1:1000), GATA6 (#5851, 1:800) and PARP (#9532, 1:1000) from Cell Signalling Technology (Lane Danvers, MA); CTNNB1 (sc-7963, 1:1000) and GAPDH (sc-25778, 1:5000) from Santa Cruz Biotechnology. Chemiluminescence was detected using Amersham^TM^ ECLTM Western Blotting Detection Reagents (GE Healhtcare).

### Immunofluorescence

H-1975 cells were seeded in 24-well plates and treated with RA for 48 and 72 hrs. At the end of the experimental endpoints, the cells were fixed in 4% paraformaldehyde for 10 min at room temperature. Cells were then permeabilized in PBS/0.2% Triton X-100 for 10 min at room temperature and blocked in PBS/3% BSA for 30 min. H-1975 cells were then incubated with primary antibody, anti- CTNNB1 (clone 6B3, 1:200, Cell Signaling, USA) over night at 4 °C. At the end of the incubation, cells were then incubated with secondary antibody Alexa 594 (1:200) from Molecular Probes (Eugene, OR, USA), for 1 h at room temperature. Cells were counterstained with Actingreen 488 (Molecular probes, Eugene, OR, USA) and Hoescht Stain Solution (1:1000) was used to label the nuclei. The preparations were analyzed by confocal microscopy (Nikon A1 Confocal Laser Microscope).

### Luciferase assay

H-1975 cells were seeded in 24 well plates and were transfected the next day with M50/M51 Super 8x TOPFlash/FOPFlash plasmid, kindly provided by Prof. Greco, Yale University. For all luciferase assay transfections pRenilla-CMV luciferase vector (Promega, Madison, WI, USA) was used as an internal transfection control. 24 hours after transfection, cells were treated with RA and, 24 hours later, luciferase readings were taken by Glomax (Promega), using the Dual-Glo luciferase assay kit (Promega), by following the manufacturer’s instructions.

### *In vivo* RA treatment of NSCLC xenografts

Five weeks old female AthymicNude-Foxn1nu mice (n = 10) were purchased from Harlan (Harlan Laboratories, San Pietro al Natisone, Italy). Animal care and handling were carried out in accordance with the EU Directive 2010/63/EU. Mice received were housed in groups of five in individual cages with *ad libitum* access to water and food (Teklad rodent diet, Harlan Laboratories). Animals were observed daily and clinical signs were noted. After an adaptation period of 10 days, each mouse was inoculated subcutaneously in the right flank with viable single human H-1975 TKI resistant NCSLC cell lines (3 × 10^6^) re-suspended 1:1 in a solution containing PBS and Matrigel (BD Biosciences) in a final volume of 0.2 ml. On day 14, when tumors were palpable, mice carrying H-1975 xenografts were randomly assigned two groups of five and were treated as follow: group 1 - PBS (vehicle Control), group 2–1 µM RA (experimental group). The day of the first RA treatment was considered as day 0. The two groups have been injected with RA or PBS twice/week till day 21 post RA injection, considered as the end of the experimental regimen. The mice from both groups were sacrificed and tumors were harvested. Specifically, we collected samples devoted to protein extraction from three xenograft, while from the other two we collected samples in RNA later devoted to RNA extraction.

### Chromatin immunoprecipitation (CHIP)

Chromatin immunoprecipitation was performed using SimpleChIP Plus kit (Agarose bead) (#9004) from Cell Signaling, according to the manufacturer protocol. Briefly, chromatin was immunoprecipitated with GATA6 antibody (#5851, 1:50) and Normal Rabbit IgG (#2729, 1:50) from Cell Signaling used as negative control. The immunoprecipitated DNA and the 2% input (DNA not immunoprecipitated) has been subjected to qRT-PCR by using primers that cover the GATA6 consensus sequences in the promoters of EGFR and CTNNB1 genes (Supplemental Table S1). Signals obtained from each immunoprecipitation were expressed as a percent of the total input chromatin, by using the established following equation^55^:

$${\rm{P}}{\rm{e}}{\rm{r}}{\rm{c}}{\rm{e}}{\rm{n}}{\rm{t}}{\rm{I}}{\rm{n}}{\rm{p}}{\rm{u}}{\rm{t}}=2\,{\rm{ \% }}\times {2}^{({\rm{C}}[{\rm{T}}]2{\rm{ \% }}{\rm{I}}{\rm{n}}{\rm{p}}{\rm{u}}{\rm{t}}{\rm{S}}{\rm{a}}{\rm{m}}{\rm{p}}{\rm{l}}{\rm{e}}-{\rm{C}}[{\rm{T}}]{\rm{I}}{\rm{P}}{\rm{S}}{\rm{a}}{\rm{m}}{\rm{p}}{\rm{l}}{\rm{e}})}.$$

### RNA interference

H-1975 cells were seeded in 6-well plates and transiently transfected with GATA6 specific small interfering RNA (siRNA). The siRNA oligos of GATA6 (L-008351-00), and the negative control siRNAs (D-001810-10-05) were purchased from GE Healthcare Dharmacon^TM^, (Lafayette, CO, USA). Lipofectamine RNAiMAX Transfection Reagent (Thermo Fisher Scientific) was used for siRNA transfection according to the manufacturer’s protocol. 24 h after transfection, cells were treated with 1 and 10 µM RA for 24 h o 48 h and harvested for RNA or protein extraction.

### Statistical analysis

All the experiments performed in the manuscript has been replicate at least three times (biological replicates) in order to perform the appropriate statistical analysis. Statistical analysis has been done by using the multiple unpaired t-test (GraphPad Prism 7.0).

## Electronic supplementary material

Supplementary Information
