# Supplementary material for: Retinoic Acid affects Lung Adenocarcinoma growth by inducing differentiation via GATA6 activation and EGFR and Wnt inhibition
Source: Sci Rep. 2017 Jul 6;7:4770. doi: 10.1038/s41598-017-05047-z (PMC5500497; doi:10.1038/s41598-017-05047-z)
Supplement: Supplementary file 1 — Supplementary Information [file 41598_2017_5047_MOESM1_ESM.pdf]

Supplementary information

Retinoic Acid affects Lung Adenocarcinoma growth by inducing differentiation via GATA6 activation and EGFR and Wnt inhibition.

Giovanni Zito[1#], Flores Naselli[1#], Laura Saieva[1], Stefania Raimondo[1], Giovanna Calabrese[2,] Claudio Guzzardo[1], Stefano Forte[3], Christian Rolfo[4], Rosalba Parenti[2] and Riccardo Alessandro[1*].

This file contains:

1. 7 Supplementary figures
2. 2 Supplementary tables

Zito, Naselli et al, Supplementary Figure S1

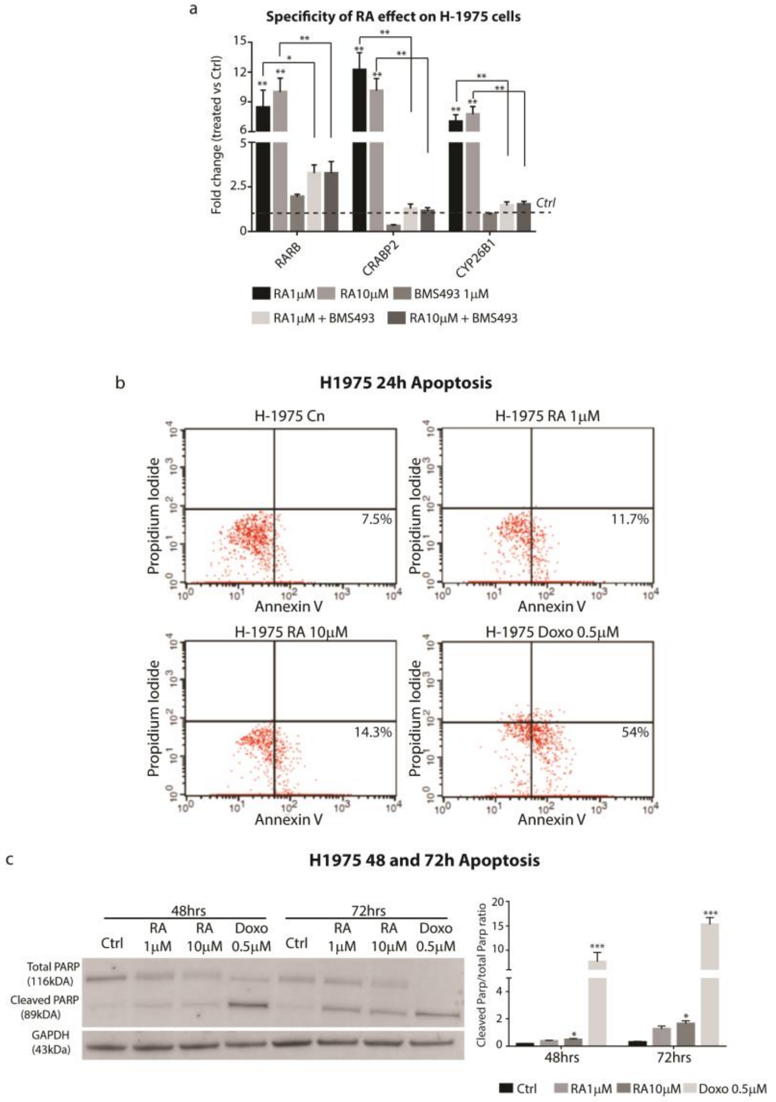

Supplementary Figure S1. Specificity of RA effect in H1975 cells and induction of apoptosis. (a) qRT-PCR analysis of *RARB, CRABP2 and CYP26B1* genes in H-1975 cells treated with 1 and 10µM RA alone or in combination with 1µM BMS493 (pan inverse RA agonist) for 48h. The comparison has been conducted by using the ΔΔCT method and normalized to *GAPDH* transcript. Dotted line represents the normalized expression levels of each transcript analyzed in untreated cells. Data are represented as mean ± SD (n=3, *= 0.02, **= 0.05). (b) Representative Annexin V/PI assay analyzed by FACS of H-1975 cells (n=3) treated with RA 1 and 10µM or 0,5 µM Doxorubicin for 24 hours. (c) Left panel**,** representative Western blotting analysis of total and cleaved PARP and GAPDH in H-1975 cells treated with 1 and 10µM RA or 0,5 µM Doxorubicin for 48 and 72 hrs. Right panel, densitometric analysis of total and cleaved PARP levels, normalized versus GAPDH, used as loading control (n=2) (*= 0.02, ***= 0.002).

Zito, Naselli et al, Supplementary Figure S2

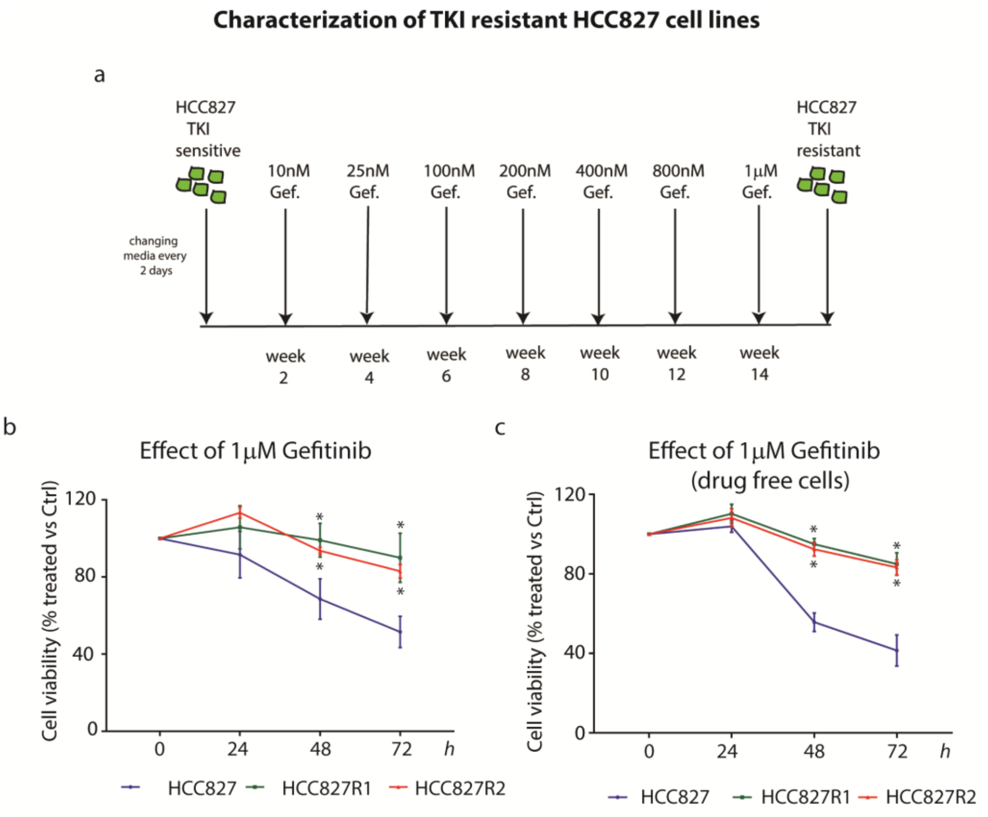

Supplementary Figure S2. Characterization of TKI resistant HCC827 cell lines. (a) Cartoon representing the experimental generation of NSCLC TKI resistant cells from HCC827 TKI sensitive. (b) Left panel, cell viability assay (MTT) of HCC827, HCC827R1 and HCC827R2 (new NSCLC TKI resistant cells) treated with 1µM Gefitinib. Right panel, MTT assay of HCC827, HCC827R1 and HCC827R2 treated with 1µM Gefitinib for 24-48 and 72hrs, a month after the end of the selection. Data are represented as mean ± SD and indicate the percentage of gefitinib treated cells versus an untreated control (n=5) (*= 0.02).

Zito, Naselli et al, Supplementary Figure S3

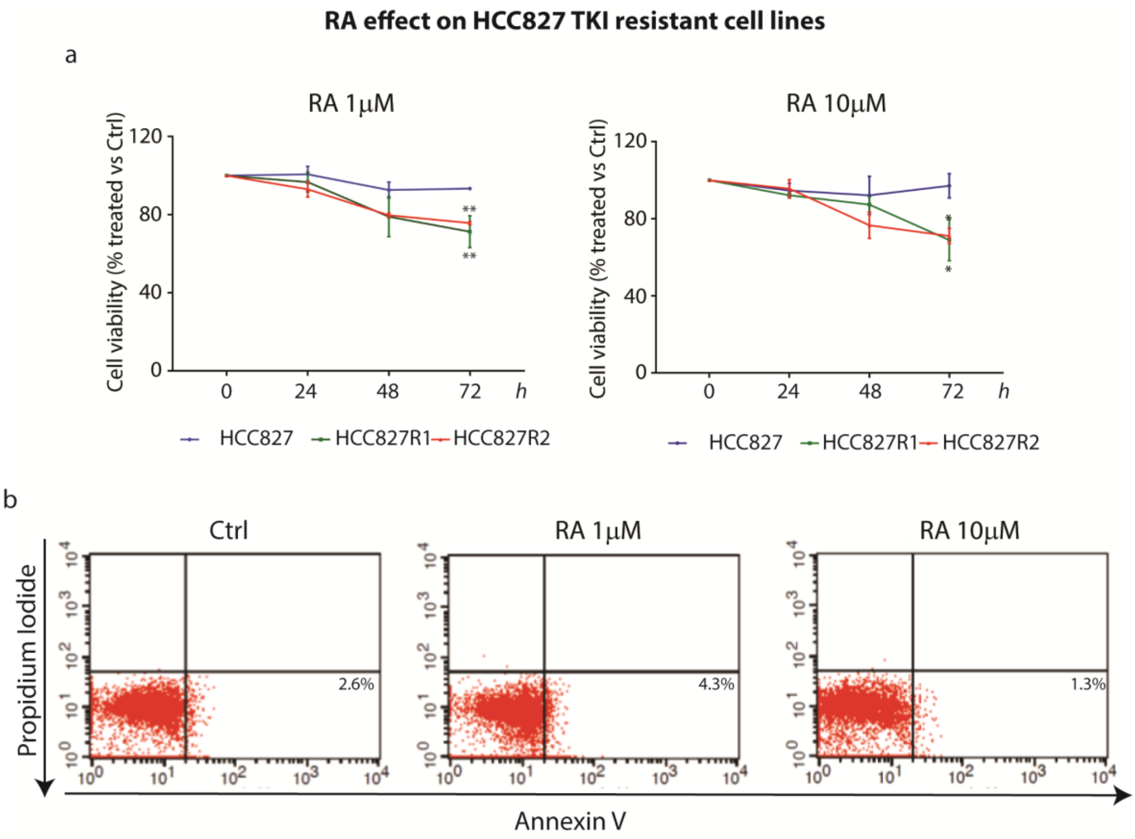

Supplementary Figure S3. RA effect on HCC827 TKI resistant cell lines. (a) Cell viability assay (MTT) of HCC827, HCC827R1 and HCC827R2 cell lines treated with RA 1 and 10µM for 24-48 and 72hrs. Data are represented as mean ± SD and indicate the percentage of RA treated cells versus an untreated control (n=3) (*= 0.02, **= 0.05). (b) Representative Annexin V/PI assay analyzed by FACS of HCC827R1 cells (n=3) treated with RA 1 and 10µM for 48h (n=3).

Zito, Naselli et al, Supplementary Figure S4

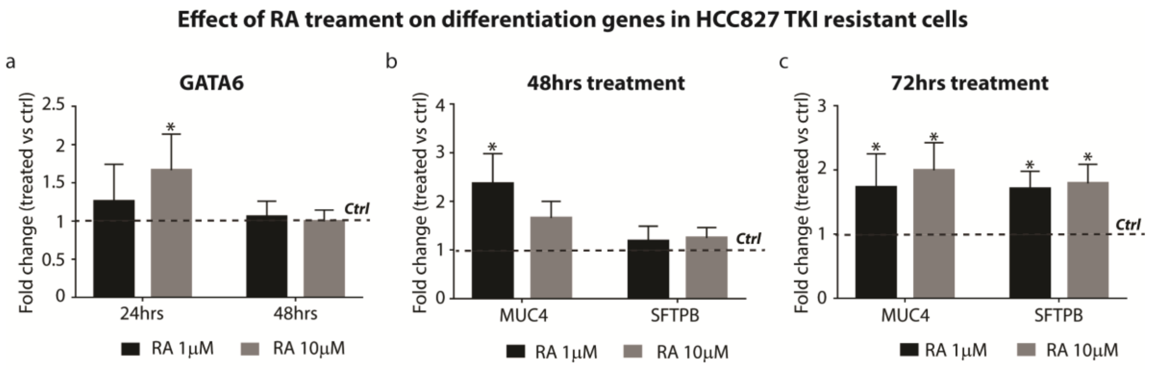

Figure S4. Effect of RA treatment on differentiation genes in HCC827 TKI resistant cells. (a) qRT-PCR analysis of *GATA6* gene expression in HCC827R1 treated with 1 and 10μM RA for 24 and 48 hrs. (b-c) qRT-PCR analysis of differentiation genes in HCC827R1 cells treated with 1 and 10μM RA for 48 and 72 hrs. The comparison has been conducted by using the ΔΔCT method and normalized to *GAPDH* transcript. Dotted line represents the normalized expression levels of each transcript analyzed in untreated cells. Data are represented as mean ± SD (n=3) (*= 0.02).

Zito, Naselli et al, Supplementary Figure 5

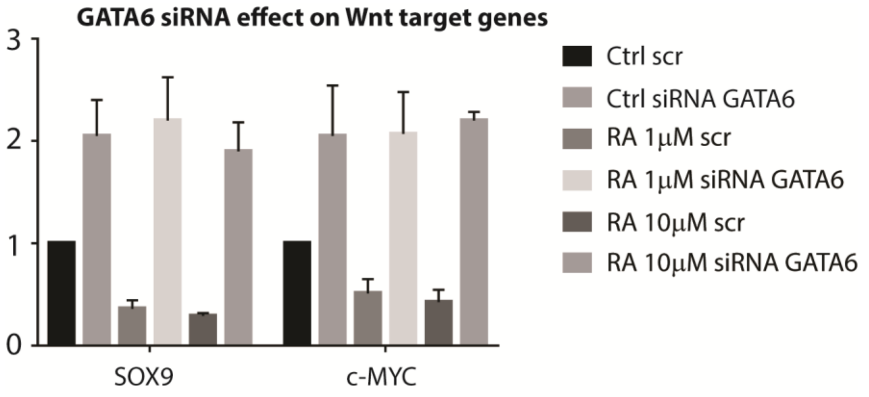

Figure S5. qRT-PCR analysis of Wnt target genes SOX9 and c-MYC after *GATA6* silencing and 1 or 10μM RA treatment for 24 hrs. The comparison has been conducted by using the ΔΔCT method and normalized to GAPDH transcript. Data are represented as mean ± SD.

Zito, Naselli et al, Supplementary Figure 6

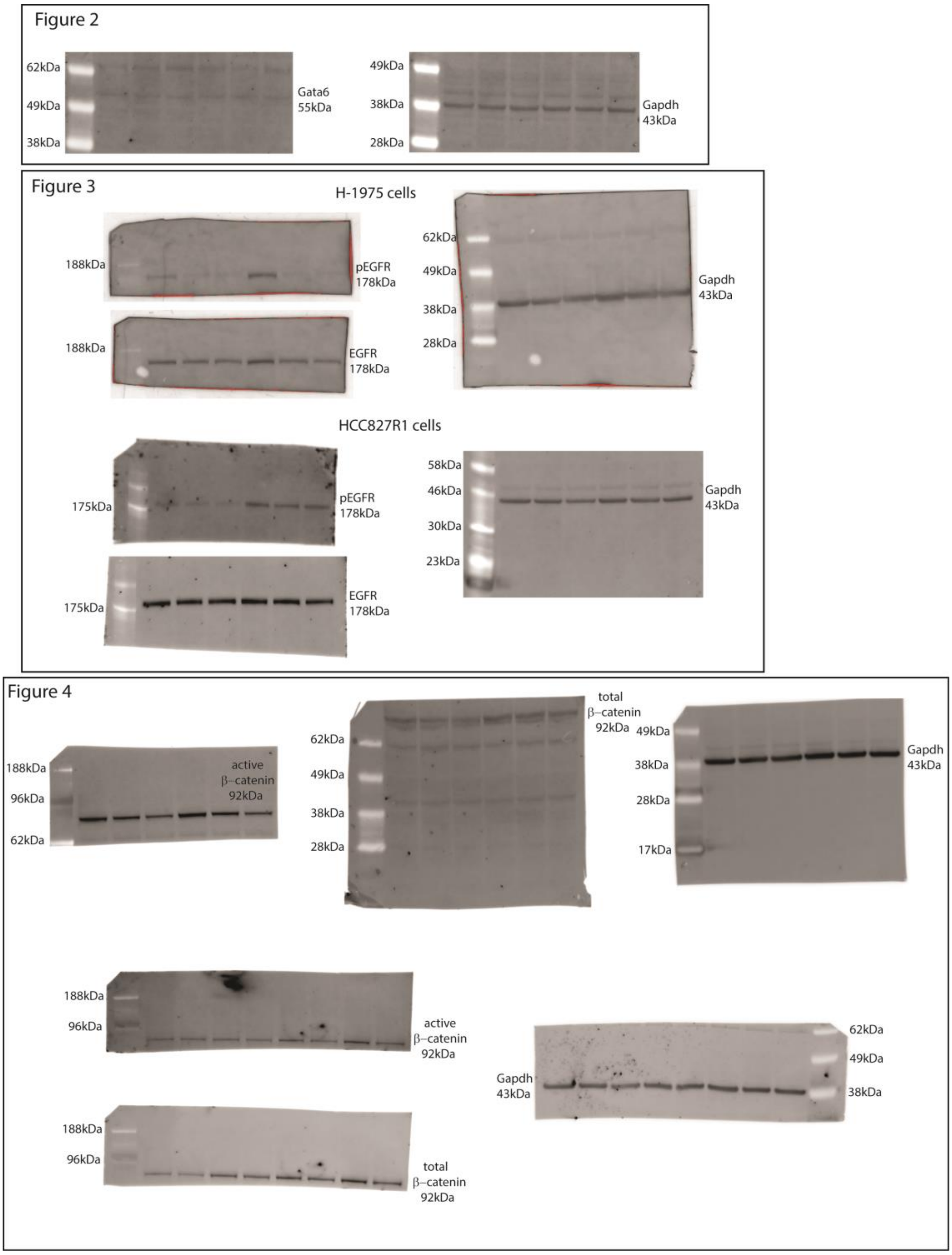

Supplementary Figure S6: Original uncropped Western Blotting reported in Fig. 2, 3 and 4.

Zito, Naselli et al, Supplementary Figure 7

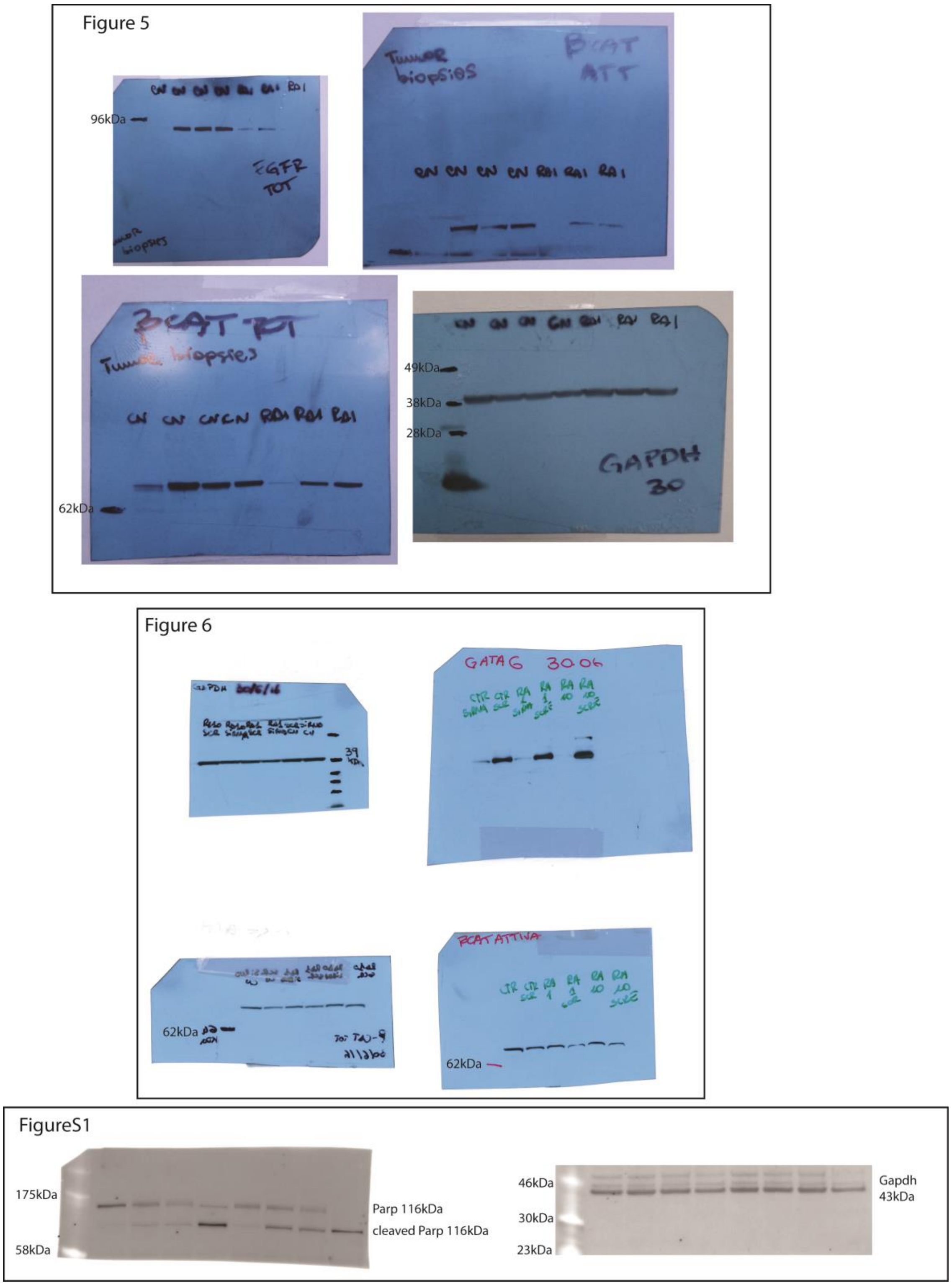

Supplementary Figure S7: Original uncropped Western Blotting reported in Fig. 5, 6 and S1.

Zito, Naselli et al, Supplementary Table S1

Supplementary Table S1. CHIP primer sequences list

Primer	Forward (5'-3')	Reverse (5'- 3')
PromEGFR1	AGAGCCAGACCCTGTCTCAA	ATCACCCAGTTTGTGGCATT
PromEGFR2	CCAGCCCCTTAGATGGTACA	TAGAAGGTCTGCCCTCAGGA
PromEGFR3	TGGATTCAAAGTTGGCCTCT	AATAGTGGCCCAGTTGCTGT
PromEGFR4	CAACGCACAGTGGCTGTAC	AATGTTTGTGCCTGGGTCTC
IntrCTNNB1	TTTGCGCCCAATATTCATTAC	ACCCCCAGGACTTCAAACTT
PromCTNNB1	AGCTGCAAGCTTTCCACAGT	TCTTTCCAGCTGAACAGGCTA
PromCTNNB2	ATTGTCCAAGGTCAATTCAAAAA	AACTTCTGTGAAATGAAGACAAACAC
PromCTNNB3	CCAAAGAAAAATCCCCACAA	CTCCAGAGCGCTGTCAATTAG

Zito, Naselli et al, Supplementary Table S2

Supplementary Table S2. qRT-PCR primer sequences list

Primer	Forward (5'-3')	Reverse (5'- 3')
c-MYC	TCCTCGGATTCTCTGCTCTC	CTCTGACCTTTTGCCAGGAG
CRABP2	TGAGGAGCAGACTGTGGATG	TCTCTGGTCCACGAGGTCTT
CYP26B1	ACACGGTGTCCAATTCCATT	GCCTCCTGGTACACGTTGAT
EGFR	CCTATGTGCAGAGGAATTATGATCTTT	CCACTGTGTTGAGGGCAATG
FABP6	GTTGGCAAGGAAAGCAACAT	TTGTCACCCACGATCTCTGA
FZD2	ATTTTTCTGTCGGGCTGCTA	CTGAAGCTGCTCCTCAGACC
GAPDH	CAATGACCCCTTCATTGACC	TTGATTTTGGAGGGATCTCG
GATA6	GGCTCTACAGCAAGATGAACG	CTGCGCCATAAGGTGGTAGT
MUC4	CAACCTCCCAGACCATCATT	GTCCTCCTGACGAACTCCAG
NKX2.1	GACGCTTCAAGCAACAGAAG	TTCATTTTGTAGCGGTGGTTC
RARB	CTCCCCCTCGAGTGTACAAA	GCATCGATTCCTGGTGACTT
SFRP4	CTCCCGGAGGATGTTAAGTG	TGCTGAGATACGTTGCCAAA
SFTBP	ACGACTACTTCCCCCTGGTC	GACGAGCTTGTCCAGCAGA
SOX9	GACTTCTGAACGAGAGCGAGA	CGTTCTTCACCGACTTCCTC
CTNNB1	GCGCCATTTTAAGCCTCTCG	CTGAAGCTGCTCCTCAGACC
